# Biocontrol Screening of Endophytes: Applications and Limitations

**DOI:** 10.3390/plants12132480

**Published:** 2023-06-28

**Authors:** Nikhil Kashyap, Sandeep Kumar Singh, Nisha Yadav, Vipin Kumar Singh, Madhuree Kumari, Dharmendra Kumar, Livleen Shukla, Kaushalendra, Nikunj Bhardwaj, Ajay Kumar

**Affiliations:** 1 Department of Biotechnology, Noida International University, Greater Noida 203201, India; nikhilkashyap907@gmail.com; 2 Division of Microbiology, ICAR—Indian Agricultural Research Institute, Pusa, New Delhi 110012, India; sandeepksingh015@gmail.com; 3 Division of Agriculture Extension, ICAR—Indian Agricultural Research Institute, Pusa, New Delhi 110012, India; 4 Department of Botany, K.S. Saket P.G. College, Ayodhya 224123, India; vipinks85@gmail.com; 5 Department of Biochemistry, Indian Institute of Science, Bangalore 560012, India; madhuree88@gmail.com; 6 C.M.B. College, Ghoghardiha, Madhubani 847402, India; dharambhu@gmail.com; 7 Department of Zoology, Mizoram University (A Central University), Pachhunga University College Campus, Aizawl 796001, India; kaushalpuc@gmail.com; 8 Department of Zoology, Maharaj Singh College, Maa Shakumbhari University, Saharanpur 247001, India; 9 Department of Botany, M.V. College, Buxar 802101, India

**Keywords:** endophytes, isolation strategies, molecular interactions, biocontrol screening, regulation and registration strategies

## Abstract

The considerable loss of crop productivity each year due to plant disease or pathogen invasion during pre- or post-harvest storage conditions is one of the most severe challenges to achieving the goals of food security for the rising global population. Although chemical pesticides severally affect the food quality and health of consumers, a large population relies on them for plant disease management. But currently, endophytes have been considered one of the most suitable biocontrol agents due to better colonization and acclimatization potential. However, a very limited number of endophytes have been used commercially as biocontrol agents. Isolation of endophytes and their screening to represent potential characteristics as biocontrol agents are considered challenging by different procedures. Through a web search using the keywords “endophytes as biocontrol agents” or “biocontrol mechanism of endophytes,” we have succinctly summarised the isolation strategies and different in vitro and in vivo biocontrol screening methods of endophytic biocontrol agents in the present review. In this paper, biocontrol mechanisms of endophytes and their potential application in plant disease management have also been discussed. Furthermore, the registration and regulatory mechanism of the endophytic biocontrol agents are also covered.

## 1. Introduction

Food security is one of the most severe concerns for the increasing global population. It has been estimated that by 2050, the global population may achieve the landmark of 9.7 billion, and consequently, there will be a need to enhance more than 50% of the existing agricultural productivity [1,2]. Further, shrinking natural resources like agricultural land, deforestation, changing climatic conditions, and loss of agricultural productivity due to pre- or post-harvest pathogen attacks or plant diseases have exerted additional pressure on securing food availability [3,4]. Plants are exposed to different biotic and abiotic stresses on a daily basis that adversely affects their growth and survivability. Approximately 15–30% of the total agricultural production is lost each year due to plant diseases or pathogen attacks [5]. However, to mitigate the challenges of plant diseases, in general, a large population relies on chemical pesticides, but the continuous and indiscriminate use of chemical pesticides adversely affects the food quality, soil texture, native microflora, and the health of human beings [4]. In addition, the contamination of soil and water ecosystem, apart from the development of pest resistance, has emerged as some of the severe challenges of using chemical pesticides. Therefore, to mitigate the challenges of biotic stress, the utilisation of microbial strains has emerged as a sustainable, eco-friendly, and non-toxic approach [5,6].

The microbial strains as antagonistic or biocontrol agents have been considered as non-toxic and a genetically stable approach, showing effectiveness against a wide range of phytopathogens, even in low concentrations [7]. In addition, the plant growth-promoting activity of biocontrol agents shows an additional benefit to the plants. During the past two decades, various microbial species, including bacteria, fungi, and yeasts, have been characterised and utilised in different parts of the world as biocontrol agents (BCAs) to control pre- or post-harvest loss of agricultural products, as well as fresh produce [3]. Endophytic microorganisms have been considered one of the most suitable approaches due to their high colonization and acclimatization potential in comparison to the epiphytic microorganism present above or outside the plant surface [8]. The multifarious plant growth-promoting activity and diversified action mode for pathogen inhibition have made the endophytes quite popular. The endophytic microbes employ various direct or indirect mechanisms during plant disease management and control of phytopathogen’s growth. Some of the important strategies involve the secretion of antimicrobial compounds and volatiles, siderophore production, competition for nutrients and space, and induced systemic resistance. Additionally, the application of biocontrol agents modulates the native microbial population, thus significantly modulating crop productivity [9]. This review paper briefly discusses endophyte biology, especially the isolation strategies and their limitation, screening methods, and action mechanisms of endophytic BCAs. In addition, the important facts from current literature pertinent to the possible application of endophytic BCAs in plant disease management have also been incorporated.

## 2. Endophytes Microbes: An Introduction

The microorganisms residing in the host plant tissue or organs without showing any apparent symptoms of infections have been considered endophytes. These endophytic microorganisms share a complex relationship with the host plant and, like other epiphytic microorganisms, directly or indirectly modulate the growth, development, and protection of the host plants from pathogen invasion [4]. Nevertheless, various factors, as well as signalling molecules, play a critical role in the host–endophyte interaction, as well as in regulating the biology and functions of endophytes [10,11].

The plants host diverse microbial communities above, outside, and inside the plant organs, and these microbes play a crucial role in the maintenance of plant health and productivity. Some of the epiphytic microbes present on the plant surface enter plant tissues and survive as an endophyte [10]. The entry of endophytes into the plant tissues is a complex process and is mediated via several signalling molecules and related proteins. Wounds and natural openings such as stomata, cotyledons, hydathodes, and the aerial sites of the root zones are the most preferred sites for microbial entry into the host tissues [11]. Although the entry of the microbial strains is facilitated by either active or passive processes, entry via the natural opening is referred to as passive, whereas the entry mediated with the help of secretory products is considered an active process [12,13].

Although the entry of microbial strains to inter- or intracellular spaces of the plant tissues is the basic first step to becoming an endophyte, however, during this process, microbes face the oxidative environment of the plant. Therefore, for successful colonization, endophytes should have the ability to produce antioxidative enzymes, such as superoxide dismutase, peroxidase, etc. For example, Balsanelli et al. [14] reported the role of antioxidative enzymes produced by *Enterobacter* spp. in the successful colonization of the poplar plant.

The effective colonization of the microbial strains is mediated through different consecutive steps [15]. For example, at the rhizospheric region of plants, roots secrete a considerable number of exudates consisting of amino acids, lipids, polysaccharides, and flavonoids, responsible for the chemotactic response of the microbe’s directing movement toward the plant surface and subsequent colonization. Noteworthy, each microbial strain is specific for the particular component of the exudates [16]. In addition to these, the colonization efficacy of the endophytes depends on various factors like plant genotypes and surrounding environment microbes’ nature, like symbiotic or pathogenic [15,17]. Studies have reported that the patterns of colonization in symbiotic and pathogenic microbes are similar to a certain extent, but the defence responses of both microbial groups were quite dissimilar. Similarly, the amount of cell wall degrading enzymes is also different in symbiotics and pathogens. For example, the amount of cell wall degrading enzymes released by the pathogens are comparatively high and elicit the host’s immune response, while the symbiotic endophytes release a small number of enzymes to a level not able to elicit the immune response [18,19,20,21].

Transmission of endophytes within the host is mediated through different modes, such as horizontal, vertical, and mixed. The horizontal mode refers to the transmission of either bacterial or fungal endophytic microbes via air, soil, and environment; however, the vertical mode refers to the transmission of endophytes via the seed, pollen, and the parent, commonly followed by bacterial species. Nevertheless, some of the microbial strains follow both the transmission modes categorised as mixed mode [15,22,23]. Studies have reported that each of the plant species harbour some endophytes during life cycle [24,25]. The composition of endophytic microbial communities generally depends on the plant genotypes, plant organs, seasons, and environmental conditions. However, in general, phyla Proteobacteria is reported as the most dominant bacterial group, followed by Actinobacteria, Firmicutes, and *Pseudomonas*, *Pantoea*, *Acinetobacter,* and *Enterobacter* are the dominant endophytic bacterial genera reported in the majority of plant or plant organs. Glomeromycota has been reported as the predominant fungal phyla, followed by Ascomycota and Basidiomycota, and arbuscular mycorrhizal fungi (AMF) have been reported as predominant endophytic fungal genera in the different host plants [15,25,26].

## 3. Manoeuvring Endophytes Isolation, Community Analysis, and Challenges

For the isolation of endophytic microorganisms, it is very crucial to remove the epiphytic microbial communities present in the plant samples. Therefore, to washout the epiphytic microorganisms, sample tissue should be treated or sterilised with various types of disinfectant, alcohol, or distilled water at a certain time interval to remove the epiphytic microbial communities. Sodium hypochlorite (NaOCl), 70% ethanol, and hydrogen peroxide (H_2_O_2_) are most often used. However, the heavy metal salts like HgCl_2_ are also used as a disinfectant to eliminate the epiphytic microflora [27,28,29]. The details of sterilisation procedures followed for the isolation of endophytes are described in Table 1.

Although the concentration of disinfectant and their incubation time depend upon the nature of plant tissues, there is some limitation to the use of chemical disinfectants. The higher concentration of disinfectants and their exposure time may cause mutagenesis, as well as the death of specific endophytes microorganisms by penetration inside the host tissue [28]. In general, the surface sterilisation protocol should be slandered in such a way that it can eliminate all the epiphytes from the sample material. However, improper sterilisation is not guaranteed for endophyte isolation. Therefore, to check the efficacy of the sterilisation protocol, the sample tissue segment is printed or rolled over suitable culture media plates [28,43]. In another method, the final washing solution is spread over nutrient-rich media plates [42]. If no growth is observed after 1–2 weeks of incubation, the surface sterilisation is considered as adequate [28,43].

The pour plate and spread plate method is the most commonly employed practice for isolating endophytic microbiota [44]. Additionally, the surface sterilised tissues (0.5 cm^2^) have been aseptically placed over suitable growth media and incubated under an optimum temperature of 28–35 °C for 2–5 days for the isolation of endophytic fungi; thereafter, emerging fungal mycelia from the tissues are transferred to the fresh media [45,46]. However, only a tiny fraction of endophytic communities are accessed through culture-dependent techniques due to unknown reasons, including the deficiency of proper minimal growth requirements [47,48,49].

Moreover, even after isolation, some endophytes do not grow properly and survive after subsequent sub-culturing under laboratory conditions [50]. This may be due to the absence of host plant-specific metabolites and residual compounds in the subsequent growth media, initially present in the pulverised plant tissues plated [50]. Therefore, the selection of growth media is essential in the isolation and culturing of the endophytes. Although, to isolate novel and rare endophytes, it is often recommended to include tiny fractions of nutrients typically available to the endophytes from the host plants in the isolation media [51]. It is possible to improve isolation [52] and the endophyte’s growth and metabolite production by adding host plant extract to the culture media [53,54,55]. Moreover, increasing sample sise, plating sise, and incubation time up to 16 weeks can also improve the isolation of rare species [51].

Recently, metagenomic approaches have been extensively used to access total endophytic diversity and community dynamics. The metagenomic study describes the community structure inside and outside of the plant tissues and provides information about the functional aspects [56]. These metagenomics approaches use the latest next-generation sequencing (NGS) technologies, 16S rRNA, 18S rRNA-based gene amplicon sequencing, and shotgun metagenome sequencing for the effective detection of both cultivable and non-cultivable microbiota, even in low abundance [57].

In the recent past, advanced next-generation sequencing (NGS) and Illumina-based sequencing have been used to study cultivable and non-cultivable microbial communities, substituting the phenotypic microarray used earlier to identify endophytes. The advancement in NGS technologies has helped considerably in understanding and describing the microbiome structure. The advancement in sequencing technologies allows a deeper and finer taxonomical investigation of the endophytic microbiome. It has also enabled the discovery of biosynthetic gene clusters of secondary metabolites that can be used to combat biotic stress [58]. In Illumina sequencing, the DNA sequence has been analysed base-by-base, thus, showing higher accuracy and providing higher phylogenetic resolution than 454 sequencings [57,59]. The metagenome generated by Illumina sequencing has given much information about and better views of functional and taxonomic diversity [60].

The advancement in sequencing technologies has also revealed the critical role of fruit microbiomes in shaping fruit resistance against pathogens and the role of host species in modulating endophytic bacterial and fungal microbiomes [61,62]. NGS performed by IGA Technology Services (Udine, Italy) using MiSeq (Illumina, San Diego, CA, USA) generated 300 bp paired-end reads and revealed that *Citrus lemon* endophytic biota was transmitted from seeds to shoots with an abundance of *Cutibacterium* and *Acinetobacter* sp. [63]. NGS sequencing of two citrus cultivars demonstrated that endophytic bacterial communities could shape different resistance against pathogens and their varied diversity according to the cultivar [64]. However, both sequencing platforms have some differences in the reading length and sequencing protocols. For instance, a study showed that analysis of the same samples by these two sequencing platforms showed that 90% of sequences overlapping and genes are highly correlated [65].

While NGS sequencing is the most commonly used technology to explore the phylogenetic relationship and species composition of a sample, information on the complete microbiome can also be obtained through whole metagenome shotgun sequencing (WMSS) [66,67], providing in-depth knowledge related to genes and functions of the microbial community [68,69]. Moreover, some limitations are also reported during the culture-independent molecular techniques; for example, primers used for the amplification of bacterial rRNA genes also amplify the mitochondria and plastid genes of the plants subsequently [70]. But, now a day, to resolve the challenges of subsequent amplification of plants and bacterial DNA locked nucleic acid (LNA), the oligonucleotide-PCR clamping technique has been employed to amplify the selected bacterial genes and reduce the chance of plant DNA amplification [71]. During analysis, per sequencing run, the 454 pyrosequencing techniques can produce roughly 1,000,000 reads; however, Illumina HiSeq 2500 can generate a high number of reads, which can correspond to 0.7 GB and over 600 GB data, respectively. Therefore, storage of a vast database of metagenomics appears as a challenging task [72]. In addition, statistical analysis and visualisation of the NGS data require appropriate computational tools and skills.

Third-generation sequencing technologies such as PacBio and MinIon are the most recent technology, detecting even single and longer reads in real time with high efficiency [59]. The combination of next-generation and third-generation sequencing methods can be used to understand genomic variations with high precision and detail, thereby providing more significant insights into the host-endophyte interaction [73]. In addition, the combined application of the latest “omics” metaproteomics and metabolomics approach will give a clear overview of endophytes [74].

## 4. Endophytes as Biocontrol Agents

In order to control phytopathogens with the least amount of environmental damage, the term “biocontrol” has been employed [75,76,77]. However, the term biological control was first used by C. F. Von in 1914 for plant disease management [78]. In general, “Biological control” is defined as the use of microbes/ genes or their products to inhibit the growth of phytopathogens or pathogen invasion [79,80,81]. BCAs are considered an eco-friendly approach used to reduce the susceptibility towards infection by enhancing the immunity of the host via eliciting immune response through the secretion of various types of bioactive compounds, including antibiotics, siderophores, and signalling molecules [82,83]. Although, utilisation of endophytic strains as BCAs has some additional benefits in comparison to other strains due to better colonization efficacy [83,84]. However, before the practical application of microbial antagonistics, various factors have been taken into account, such as the survivability and stability of BCAs inside the host, as well as against the pathogens serving as the prime factors. In addition, these factors also play a significant role in practical applications and marketability [4]. In the laboratory trial, either in vivo or in vitro, a number of endophytic and epiphytic strains, including bacteria, fungi, and yeast, showed antagonistic potential, but very few microbial BCAs have been used for commercial purposes. The lack of stability and survivability of BCAs are the prime reason for such failures. But still, very limited microbial strains, particularly those belonging to the species of *Bacillus* and *Trichoderma,* have been commercialised. The endospore-forming capability of these two microbial groups has made them a popular candidate of choice due to better stability and survivability compared to other non-endospore-forming microbial strains [4].

In the last two decades, researchers have made significant progress in the commercialisation of BCAs, and these microbial antagonistic have been used in different parts of the world for the effective management of pre- as well as post-harvest loss of fresh produces. However, after screening, implementation of BCAs against the pathogen and the plant largely depends upon the host immune system, signalling molecules, metabolic activities, and then, most importantly, the growth stage of the pathogen. In addition to the genetic constitution, cultivars and physiology of plants also play a crucial role in the successful application of BCAs [85]. Although environmental conditions also affect the interaction of host–pathogens–BCAs, and thus need to be standardised for the optimum effectiveness of biocontrol applications [86,87].

## 5. Screening of Bio Control Agents

### 5.1. In Vitro Screening

Plants host numerous microbial communities as epiphytes and endophytes; some of them protect the plants and fruits from pathogen invasion by inhibiting pathogen growth. Therefore, the selection of microbial strains is the primary step of biocontrol screening [88]. Further, strains are characterised on the basis of several parameters like the synthesis of antibiotics, enzymes, bioactive compounds, siderophore productions, volatiles emission, etc. [89,90,91].

Dual culture methods are one of the simple and primary methods employed for in vitro screening of BCAs. The key advantage of dual plate assay is the quick and highly reproducible results, and their results are easily accessible and quantifiable through measurement of the inhibition zone and measurement of spore germination [92,93]. In addition, microbial synthesised antimicrobial/bioactive compounds can be easily isolated, quantified, and identified through the latest techniques. However, some limitation of dual plate culture assays is also reported during the experiment. For example, the secretion of volatiles, including the secondary metabolites, is directly influenced by the nutrient’s composition and the stage of microbial growth. In addition, the synthetic nutrients media are highly rich in comparison to the soil and the natural media. Thus, the results of the dual plate culture test are beyond reality to a certain extent [94]. However, during in vitro screening, different methods have been employed to screen the biocontrol agents:

Dual Culture Assays: In this assay, the biocontrol agent and the plant pathogen are co-cultured in vitro, either on solid agar media or in liquid culture systems. The inhibition of pathogen growth or the reduction of disease symptoms in the presence of the biocontrol agent are assessed [95].

Antagonism Assays: Through this assay, the biocontrol agent is tested for its ability to inhibit the growth or development of plant pathogens through direct antagonism by using different mechanisms like inhibition zone assays, confrontation assays, or growth inhibition measurements [96].

Enzymatic Assays: By measuring the secreted enzymes of biocontrol agents like chitinase, glucanase, protease, or cellulase production biocontrol activity of any endophytic microbial stains can be assessed. As these enzymes are directly involved in the degradation of pathogenic compounds or suppression of pathogen growth, the potential of biocontrol agents can be evaluated [97].

Evaluation of volatile compounds: The evaluation of volatiles released by the microbial antagonistic is another common method of biocontrol screening. During the analysis, both the microbial antagonistic and pathogens are grown on the agar plate separately, and the plates are sealed with parafilm to avoid the diffusion of volatiles [98].

### 5.2. In Vivo Method

In vivo method of biocontrol screening is considered one of the authentic approaches. During the in vivo analysis, the microbial antagonistics and the pathogens are inoculated into the fruits/ plant parts. Further, by measuring the lesion diameter, the severity of the diseases has been assessed to determine the effectiveness of the biocontrol agent. Although, during the inoculation, either the pathogens or microbial antagonistic are inoculated at fixed time intervals [95,99].

Seedling Growth Assays: through this assay biocontrol efficacy of the endophytic strains have been evaluated during seedling germination or seedling growth, and their impact has been examined by measuring the rate of germination, root length, shoot length, and overall seedling vigour [100].

Foliar Pathogen Inoculation Assays: Through this assay, the efficacy of the biocontrol agent has been evaluated after application to the plant leaves, followed by inoculation with a specific plant pathogen. The progression of disease symptoms, such as lesion development or disease severity, is monitored to assess the biocontrol efficacy [101].

Root Drench or Soil Application Assays: Through this method, a biocontrol agent is applied to the soil or directly to the roots of plants. Furthermore, the pathogens have been inoculated either by soil inoculation or root colonization. Then, after measuring disease incidence, severity, or pathogen population, dynamics are measured to evaluate the biocontrol efficacy [102].

Greenhouse and Field Trials: Greenhouse and field trials involve the evaluation of microbial biocontrol agents under realistic conditions. Plants are treated with the biocontrol agent, and their growth, disease incidence, yield, or other relevant parameters are assessed over a specific period in a controlled or field setting [102].

**Figure 1:**
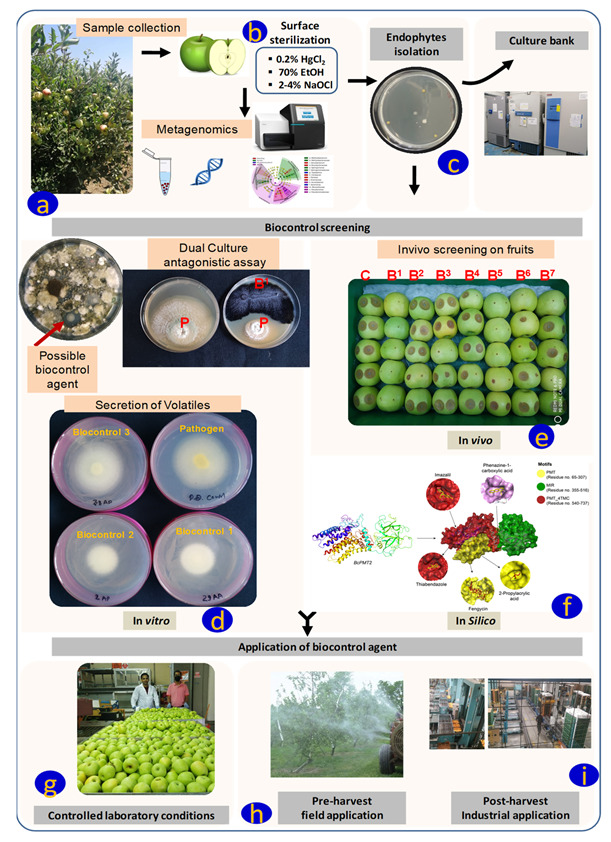
Schematic representation of biocontrol screening and their potential application; (**a**) sample collection for endophyte isolations; (**b**) surface sterilisation to remove epiphytic microflora; (**c**) endophyte isolation; (**d**) biocontrol screening; in vitro; possible biocontrol agent: clear zone around the bacterial colony can be considered for future biocontrol agent, dual culture antagonistic assay; strain B^1^ inhibits the growth pathogen; secretion of volatiles: biocontrol agent 1 and 2 reduce the pathogen growth by volatile synthesis; (**e**) in vivo- strain B^1^(Bacteria^1^) can be considered as biocontrol agents because the strain completely inhibits pathogen growth in comparison to (C) control (fruits with pathogen only); (**f**) in silico- approach of biocontrol screening; (**g**) application of biocontrol agent in controlled laboratory conditions; and (**h**,**i**) pre- and post-harvest application of biocontrol agents. P means pathogen, B2–B7 means Bacteria 1 to 7.

In addition, during in vivo analysis, several other factors like water content, oxidative stress, and other physiological statuses of the fruits and plants are also considered to achieve effective BCAs and their effect on plants [103]. The detailed method of endophyte isolation from fruit sample, sterilisation, screening of BCAs, and their application in post-harvest management is described in Figure 1.

## 6. Biocontrol Mechanisms of Endophytic Microorganisms

### 6.1. Induced Systemic Resistance (ISR)

Plants possess various biochemical and physiochemical mechanisms to cope with biotic and abiotic stresses. The plant releases various types of signal molecules during stress conditions to regulate metabolic behaviour and enzymatic reaction, enhancing the resistance against the stresses. Although in case of biotic stress, such as pathogen invasion, the plant system protects itself via enhancing resistance through an immune response. The induction in resistance can either be systemic acquired resistance (SAR) or induced systemic resistance (ISR). The SAR is induced by the locally infected tissue resulting from the attack of necrotizing pathogens and spreads to the plants via signalling molecules [104]. However, the ISR enhances the resistance of the whole plant against the attack of various pathogens. Usually, ISR is induced by the BCAs [104]. However, induction of the plant defence system is mediated via signalling molecules and is recognised by specific receptors like PRRs and proteins related to pathogenesis [105]. Different authors have reported the potential of endophytic microbes in eliciting the ISR through different mechanisms like induction of pathogenic protein synthesis and modulation of signalling pathways in response to biotic stress [106,107,108].

### 6.2. Competition for Nutrient and Space

In the quadritrophic system involving fruits, pathogens, and endophytes, the host faces limitations in terms of nutrition and space. Both endophytes and pathogens rely on essential nutrients such as nitrogen, carbon, macro, and micronutrients for their survival [4]. However, after the application of microbial biocontrol agents, these agents compete with the target pests or pathogens for space and nutrients by colonizing the same ecological niches or habitats as the target organisms, such as plant surfaces, soil, or water, and trying to establish themselves and proliferate. The biocontrol agents can outcompete the pests or pathogens by utilizing available resources more efficiently, depriving them of essential nutrients, or occupying physical spaces that prevent their establishment. In addition to competing with the target organisms, microbial biocontrol agents may also compete with the native microbial communities [4]. Native microorganisms are naturally present in ecosystems and play important roles in nutrient cycling, disease suppression, and maintaining ecosystem balance. The introduction of biocontrol agents can disrupt the existing microbial communities and create competition for resources [109]. For example, Oszust et al. [110] reported elimination of pathogen *Colletotrichum* spp. after applying the *Trichoderma* genus due to fast colonisers and nutritional competition. In a study, Hernandez et al. [111] reported the inhibition of pathogen spore germination by the endophytic bacterial strain *Bacillus* sp. through the rapid utilisation of carbon sources. Similarly, Lappa et al. [112] have reported the biocontrol activity of *Lactobacillus plantarum* against the pathogen *Aspergillus carbonarius*. In vitro studies showed higher colonization efficacy of the biocontrol agents. In addition, the transcriptomic study also showed that co-culturing of biocontrol agents *Lactobacillus plantarum* with pathogen *Aspergillus carbonarius* significantly enhanced the expression of the nutrient uptake gene, which indicates the competition for nutrients between *Lactobacillus plantarum* and pathogen *Aspergillus carbonarius*. However, the outcome of the competition for space and nutrients after microbial biocontrol application depends on various factors, including the characteristics of the biocontrol agents, the target organisms, and the environmental conditions. Some biocontrol agents may have traits that give them a competitive advantage over the target organisms, allowing them to establish and persist in the ecosystem. However, in some cases, the biocontrol agents may struggle to compete effectively, leading to limited success in pest or pathogen control.

### 6.3. Defence Enzymes

Syntheses of defence enzymes by the endophytic microorganism are one of the common mechanisms of phytopathogen control [113]. Like other epiphytic microorganisms, endophytes secrete different defence enzymes, such as chitinase, β-1,3-glucanase, peroxidase, etc. [113,114], which mediate their role in pathogen growth inhibition. For example, the enzyme chitinases degrade the chitin, one of the important constituents of the pathogen cell wall, and inhibit the pathogen growth. Different authors have reported the role of chitinases in pathogen control. Quecine et al. [115] reported the role of chitinases secreted by *Streptomyces* in *Colletotrichum sublineolum* growth inhibition. Similarly, Bressan and Figueiredo [116] have reported the contribution of chitinases produced by *Bacillus* spp. In the growth inhibition of *Fusarium moniliforme* in the maise crop.

Rajendran et al. [117] reported consortia of different microbial strains, namely *B. subtilis*, *P. fluorescens*, and *Trichoderma viride* in the effective management of *Ganoderma lucidum* (Leys), in coconut palm the defense enzymes such as peroxidase, polyphenol oxidases (PPO), phenylalanine ammonia-lyase (PAL) synthesis. Similarly, Abdel-Rahim and Abo-Elyousr [118] reported that enzymes released by *Talaromyces acidophilus* significantly inhibit the growth of *Botrytis cinerea* growth. Cao et al. [119] reported the growth inhibition of pathogen *Pythium* sp. by β-1,3-glucanases synthesised by the *Choiromyces aboriginum*, thus playing a crucial role.

### 6.4. Antibiosis

Microbes secrete a large number of bioactive compounds, including the antimicrobial metabolites broadly utilised in pharmaceutics, agriculture, and healthcare. Although antimicrobial compounds are of low molecular weight, heterogeneous compounds are produced by a range of microbes, including *Pseudomonas*, *Bacillus*, *Agrobacterium*, *Bacillus*, *Pantoea*, *Streptomyce*, and *Trichoderma* [120]. Various authors have briefly reviewed the bioactive compounds secreted by endophytes and how they effectively manage phytopathogen growth and plant disease management [121,122,123].

The Synthesis of antimicrobial compounds by the biocontrol agent is one of the most promising approaches to phytopathogens growth. A study has reported the production of antimicrobial compounds by microorganisms [124]. The most important metabolites synthesised by *Bacillus* genera are lipopeptides, iturin, and surfactin. Similarly, *Pseudomonas* synthesises metabolites like DAPG, pyrrolnitrin, and phenazine [45]. The different *Trichoderma* species are known for the production of some specific antibiotics like clonostachys, gliovirin, gliotoxin, and viridin [125]. These secreted metabolites are well-known antibiotics showing inhibitory effects on the phytopathogens in vivo or in vitro conditions. The production of antimicrobial compounds depends on several factors, including the growth stage, media, nutrient compositions, etc. The author has also reported that the production of some antibiotics depends upon a specific microbial population or by reaching a particular threshold.

### 6.5. Siderophores Production

Iron “Fe” is one of the crucial elements required for the growth and development of both biocontrol agents and phytopathogens. Some of the endophytic microbial strains produce significant amounts of siderophores. These siderophores form a stable complex with available iron, limiting its bioavailability, especially on the exposed surface of the host plant. In the competition for survival, endophytes have an advantage over pathogens as they can quickly colonise the fruit and produce various types of siderophores, effectively chelating and sequestering iron [4]. This deprivation of iron sources hampers the pathogens’ ability to thrive. Research has identified specific endophytes that produce siderophores and offer protection against various phytopathogens. For instance, genome mining has unveiled the production of siderophores by the endophytic *Pseudomonas fluorescens* BRZ63, which confers protection against post-harvest pathogens like *Colletotrichum demotion*, *Sclerotinia sclerotiorum*, and *Fusarium avenaceum* [126]. Certain endophytic *Bacillus* species are also known to produce a particular type of siderophore called bacillibactin, which helps safeguard bananas against bacterial wilt. Another example is *Trichoderma* sp., which produces hydroxamate siderophores capable of depleting the level of iron and inhibiting pathogen growth, briefly reviewed by Carmona-Hernandez et al. [111]. However, to enhance the efficiency of endophytes in phytopathogen growth or pathogen invasion, it is important to optimise the concentration of endophytes and consider the factors that influence siderophore production [127].

## 7. Application of Microbial Endophytes in Plant Disease Management

Plant disease management using endophytic microorganisms is an emerging aspect. Although the extent of endophytic microbes’ application as BCAs is very low in comparison to epiphytic microorganisms. However, various authors have utilised different endophytic microbes, including bacteria, fungi, including yeast, as BCAs. Li et al. [128] evaluated the biocontrol potential of endophyte *B. velezensis* isolated from maise against the pathogen *K. pneumoniae* and found significant control of the pathogen. In a study, Doty et al. [129] evaluated the biocontrol potential of *Burkholderia vietnamiensis* against a range of pathogens such as *Rhizoctonia solani*, *Fusarium culmorum*, *Gaemannomyces graminis* var. *tritici* (Ggt) and *Pythium ultimum* and reported effective management by the biocontrol agent. Wang et al. [130] reported inhibition of *Nigrospora sphaerica* in the passion fruit after the application of the endophytic bacterial strain of *Bacillus subtilis.* Similarly, Malvi et al. [131] reported the biocontrol potential of *Bacillus subtilis*, *B. pumilus*, *Burkholderia cepacia,* and *Enterobacter kobei,* against the pathogen *P. nicotianae* in the pot experiment and found effective management. In a study, Shi et al. [132] evaluated the biocontrol potential of *Pseudomonas putida* biovar, which showed effective management of the pathogen *Phytophthora nicotianae*, and strain also showed strong colonization efficacy. Further, Furuya et al. [133] found the effective antagonistic potential of *Bacillus subtilis* against the pathogen *Botrytis cinerea* and *Colletotrichum gloeosporioides.* Nian et al. [134] reported the effective biocontrol potential of *Vishniacozyma victoria* against the pathogen *Botrytis cinerea*. In a study, Xia et al. [135] reported the antagonistic activity of the endophytic bacterial strain *Bacillus subtilis* against the pathogen *Colletotrichum fructicola*, and their possible mechanism of action was the secretion of bioactive metabolites, defense enzymes, and siderophores production. Similarly, in another study, Ma et al. [136] reported a significant reduction in kiwi canker diseases after the application of antimicrobial metabolites isolated from the endophytic fungal strain *Fusarium tricinctum.* Vijitrpanth et al. [137] manage the panicle disease of rice after the application of endophytic *Trichoderma* sp. Pushpalatha et al. [138] reported significant control of the pathogen *Sclerospora graminicola* after the application of *Streptomyces* spp. These studies showed the potential application of endophytic microbial strains for plant disease management or phytopathogen control.

## 8. Registration and Regulation of Microbial Bio Control Agents

Microbial biocontrol agents typically require registration or authorization before they can be marketed and used. For this, the various Regulatory authorities, such as the Environmental Protection Agency (EPA) in the United States or the European Food Safety Authority (EFSA) in the European Union, oversee the registration process and are the point of contact for the registration process [139]. This process includes a series of events like Labelling and Documentation: Proper labelling of microbial biocontrol agents is necessary, including information on product identity, instructions for use, safety precautions, and storage conditions. In addition, documentation, including data on safety, efficacy, and quality, is typically required for regulatory submissions. Post-Market Surveillance: Once microbial biocontrol agents obtain approval from the authority and are distributed in the market, post-market surveillance is often conducted to monitor their continued safety and effectiveness. However, the specific safety and regulatory requirements may differ among countries and regions. It is advisable to consult the regulatory authorities for detailed guidelines and specific requirements related to the safety and registration of microbial biocontrol agents [140,141].

## 9. Microbial Biocontrol Agents such as GRAS and QPS Organisms

GRAS (Generally Recognised as Safe) organisms are considered safe for human consumption or during their common practices. Therefore, for commercial purposes, the registration of microbial biocontrol agents as GRAS organisms has been declared mandatory by different authorities [142,143]. Similarly, the term “QPS organisms” refers to microorganisms that are considered Qualified Presumption of Safety by the European Food Safety Authority (EFSA). These microorganisms have a history of safe use and are presumed to be safe for their intended purpose [144,145]. Although recognition of GRAS and QPS organisms varies among different countries, due to different jurisdictions, and regulatory authorities. However, a certain procedure has been followed to declare any microorganisms or biocontrol agents as GRAS and QPS organisms [146].

Proper taxonomic identification and characterization: The strain must be accurately identified and classified up to species or subspecies level, using appropriate taxonomic methods, such as DNA sequencing and phylogenetic analysis. Further strains have been extensively characterised on the basis of morphology, physiology, and biochemical characteristics. Safety assessment: The safety assessment of the tested biocontrol strains is mandatory to demonstrate that the strain does not pose any risk to human health, animals, or the environment; Efficacy evaluation and action mechanisms: The biocontrol efficacy of the strain against the target plant pathogens or pests needs to be demonstrated through scientifically rigorous experiments. This includes in vitro and/or in vivo studies that show the ability of the strain to control or suppress the target pathogens or pests. Furthermore, the details action mechanisms of the strains have been critically evaluated.: Environmental impact assessment: An assessment of the potential environmental impact of the strain is necessary to ensure that its introduction and use as a biocontrol agent will not have adverse effects on the environment or non-target organisms; Production and quality control: Information about the production process, quality control measures, and stability of the strain is required to ensure consistent production and quality of the biocontrol agent [147,148].

## 10. Conclusions and Future Prospective

In the recent past, endophytic microorganisms have gained popularity as BCAs due to their excellent colonization efficacy and better acclimatizing potential. In addition to the effective management of phytopathogen, the multifaceted approach of endophytes in growth promotion via phytohormone modulations, phosphate solubilisation, and siderophore productions. Furthermore, they provide an extra benefit in penetrating the plant tissues, colonizing inside the host, and efficient transmission to the next generation of plants through seeds in comparison with the rhizospheric microbiota [149]. Screening of compatible microbes which can colonise inside host tissues and can be transferred to the next generation of the plants via seeds can be an innovative approach to obtain the maximum benefits from endophytes in plant disease management [150]. Similarly, the interaction of screened endophytes with the plant microbiota should be researched in detail to obtain an in-depth idea of the desirable and undesirable consequences of endophytic application in plant microbiomes.

Screening criteria play a substantial role in isolating potential endophytes. Many endophytes such as *Trichoderma* sp., *Bacillus* sp., and *Pseudomonas* sp. are routinely screened via standardised isolation techniques. However, it is imperative to modify the screening conditions to isolate endophytes that can tolerate biotic and abiotic stress conditions and later validate them in different environmental conditions [151]. Further, recent advances, NGS technology, and omics approaches can help in isolating and screening the non-culturable endophytes and help in elucidating the plant-pathogen-endophyte interactions at a molecular level. Moreover, a global, country-specific database of isolated endophytes and their metabolites can be prepared, which can serve as a roadmap for further screening, identification, and application of endophytes in plant disease management [152].

Fungi and bacteria are the most frequently screened endophytes in plant disease management; however, recent studies have shown an important role of endophytic actinomycetes and yeasts in plant disease management [153,154]. More studies on unconventional types of microbes as endophytes are required for screening a better endophytic candidate for tolerating biotic stress in plants.

Despite the multiple benefits of endophytes in plant disease management, their uses in field conditions are limited. More focus should be provided on screening stress-tolerant endophytes and their translation from lab to land. Furthermore, initiatives should be taken to educate the end users on this technology for efficient and environmentally friendly plant disease management.

## Figures and Tables

**Table 1:** Latest sterilisation procedures followed during endophyte isolation.

Endophytes	Plants Part/s	Sterilisation Technique	References
Fungi	*Andropogon gerardii* plant tissue	Samples tissue washed with 75% ethanol for 1 min, followed by 50% commercial bleach and 75% ethanol for 1 min, and lastly with sterilised water for 1 min.	[30]
Fungi	Seeds of the common bean	Sample tissues washed with 96% ethanol and 1% NaOCl for 10 s and 3 min, respectively, three times and finally washed with sterilised water.	[31]
Fungi	Leaves of *Ephedra pachyclada*	Initial washing with sterilised distilled water for 1 min followed by 70% ethanol for 1 min, 2.5% NaOCl for 4 min, then rewashed with 70% ethanol for 30 s, and sterilised water.	[32]
Fungi	Twigs of Scots pine	Surface sterilisation by dipping for 1 min into 70% ethanol, followed by 2% NaOCl for the same time, and finally washing with sterilised water.	[33]
Bacteria	Leaves of *Arabidopsis thaliana*	Surface sterilisation by 75% ethanol for 1 min, followed by bleaching with 5% (NaClO) for 1 min	[34]
Bacteria	Tomato fruits	Washing with 75% ethanol for 20 min, followed by sonication and washing with 2.7% bleaching agent for 20 min.	[35]
Bacteria	Tomato roots	Surface sterilisation by soaking in 3% bleaching agent for 5 min	[36]
Bacteria	Roots and leaves of halophytes	Use of ethanol (75% *v*/*v*) for 60 s followed by 3% *w*/*v* (NaClO) for 10 min, followed by ethanol (75% *v*/*v*) for 60 s, and further washed 10 times with sterilised distilled water.	[37]
Bacteria and fungi	Roots and leaves of *Arnebia euchroma*	Sample was washed for 2 min in 70% (*v*/*v*) ethanol, followed by 1% (*v*/*v*) NaClO for 1 min, then again in 70% (*v*/*v*) ethanol for 2 min.	[38]
Bacteria	Stems and roots of *Salicornia europaea* L., *Salsola australis* (R.Br.), and *Bassia sedoides* (Pall.)	The samples were washed firstly with tap water, then kept in 70% ethanol solution (roots for 10 min and 15 min for stems). Further samples were placed in an 18% H_2_O_2_ solution for a fixed time interval (roots for 15 min and 20 min for stems). Then, the samples were washed four times (3 min) with sterilised water.	[39]
Bacteria	Roots of *Salicornia europaea*	Roots samples were washed with 70% ethanol for 2 min, followed by washing with sterile 2% NaCl (three times), sterilised with 15% of H_2_O_2_ for 5 min, and then washed three times with sterile 2% NaCl.	[40]
Bacteria	Root and shoot of *Triticum aestivum*	Samples were washed with 0.1% HgCl_2_ for 2 min, followed by 70% ethanol for 60 s; further washed with sterilised distilled water.	[41]
Fungi	Stem and leaves of *Ziziphus nummularia*	Samples were washed with 0.1% HgCl_2_ for 1 min, and were further washed with sterilised water for 1 min.	[42]

## Data Availability

Not applicable.
